# Dosimetric Comparison of One-, Two-, Three-, and Four-Arc Volumetric Modulated Arc Therapy Plans in Cervical Carcinoma: Evaluation of Plan Quality, Organ-at-Risk Sparing, and Treatment Efficiency

**DOI:** 10.7759/cureus.116574

**Published:** 2026-09-20

**Authors:** Priyadharshini G, Himanshi Khattar, Piyush Kumar

**Affiliations:** 1 Department of Radiation Oncology, Shri Ram Murti Smarak Institute of Medical Sciences, Bareilly, IND

**Keywords:** cervix carcinoma, plan quality, radiotherapy, treatment efficiency, vmat

## Abstract

Background

Volumetric modulated arc therapy (VMAT) has transformed the delivery of pelvic radiotherapy in cervical carcinoma, enabling precise dose conformity around critical organs while maintaining efficient treatment delivery. One important planning consideration that remains insufficiently explored is the optimal number of arcs required for achieving the best dosimetric outcome. This study was designed to address that question by directly comparing one-, two-, three-, and four-arc configurations within the same patient cohort.

Methods

Eleven patients with cervical carcinoma receiving external beam radiotherapy to 50 Gy in 25 fractions were included. Four separate VMAT plans were generated for each patient on the Eclipse treatment planning system (version 17.0.1, Varian Medical Systems, Inc., Palo Alto, CA, USA), differing only in the number of arcs employed. Plans were assessed for conformity index (CI), homogeneity index (HI), monitor unit (MU) output, and dose to the bladder, rectum, bowel, pelvic bone marrow, and bilateral femoral heads.

Results

Target conformity did not differ significantly across arc configurations (median CI, 0.83 for all arcs; Friedman X^2^ = 6.0, p = 0.112). Dose homogeneity improved significantly with increasing arc number (median HI, 0.09 (one arc) to 0.06 (four arcs); Friedman X^2^ = 18.692, p < 0.001), with Holm-adjusted post-hoc comparisons confirming significant differences for one-arc vs. four-arc (p = 0.034) and two-arc vs. four-arc (p = 0.037). MUs and bone marrow Dmean also changed significantly with arc number (MU: X^2^ = 22.056, p < 0.001, with one-arc vs. three-arc, one-arc vs. four-arc, and two-arc vs. four-arc showing significant post-hoc differences; bone marrow Dmean: X^2^ = 16.879, p < 0.001, with one-arc vs. four-arc and two-arc vs. four-arc showing significant post-hoc differences). Bladder D100%, bowel D195cc, planning time, and bilateral femoral head doses showed a significant overall Friedman effect; of these, only the bladder D100% (three-arc vs. four-arc) pairwise comparison remained significant after Holm correction. Bladder 50%, rectum D50%, rectum 100%, bowel D100%, and CI showed no significant overall Friedman effect.

Conclusions

Increasing arc number significantly improves dose homogeneity and reduces bone marrow dose at the cost of a significant increase in MUs, without compromising target conformity. Reductions in bladder, bowel, and femoral head dose, and changes in planning time, showed an overall significant arc-number effect but could not be pinned to specific pairwise comparisons after correction, with the exception of bladder D100% (three-arc vs. four-arc). Four-arc plans, and to a lesser extent, three-arc plans, offer the most favorable dosimetric profile and may be considered preferred configurations for pelvic VMAT in cervical cancer, pending confirmation in larger prospective studies.

## Introduction

Cervical cancer remains a major health burden worldwide and is one of the most common malignancies affecting women globally, particularly in developing countries [1]. In India, cervical carcinoma is the second most commonly diagnosed cancer among women and contributes significantly to cancer-related morbidity and mortality [2]. Advances in radiotherapy techniques have significantly improved target dose conformity and organ-at-risk (OAR) sparing. Modern radiation therapy techniques include brachytherapy boost treatment [3], intensity-modulated radiation therapy (IMRT), and volumetric modulated arc therapy (VMAT) [4]. VMAT has emerged as an advanced treatment modality for delivering highly conformal radiation doses while minimizing exposure to surrounding normal tissue [5].

Over the years, advances in radiation therapy techniques have significantly improved target dose conformity and OAR sparing. VMAT is delivered using a linear accelerator (LINAC), in which the gantry rotates around the patient while they lie on the treatment couch. During treatment delivery, dose rate, gantry speed, and multi-leaf collimator (MLC) movement are dynamically varied to achieve optimal dose distribution [6]. Compared with conventional IMRT techniques, VMAT has been shown to reduce treatment delivery time and monitor units (MU) while maintaining comparable or superior target coverage and OAR sparing [7].

Researchers in several studies have evaluated the dosimetric advantages of VMAT in cervical carcinoma and demonstrated improvements in conformity index (CI), homogeneity index (HI), and reductions in OAR doses compared with conventional radiotherapy techniques [5,7]. However, few researchers have specifically investigated the impact of varying the number of arcs in VMAT planning for cervical cancer. The number of arcs used in VMAT planning may influence target coverage, dose homogeneity, CI, and MU efficiency. Increasing the number of arcs may improve dose modulation and dose distribution, although it may also increase planning complexity and treatment time [7].

Therefore, the present study was designed to compare one-arc, two-arc, three-arc, and four-arc VMAT plans in cervical carcinoma. We evaluated dosimetric parameters, including CI, HI, OAR doses, MU efficiency, and planning or treatment time, to determine the optimal arc configuration for pelvic VMAT in cervical carcinoma. The primary endpoint was dose HI, and secondary endpoints were CI, OAR doses (bladder, rectum, bowel, pelvic bone marrow, and bilateral femoral heads), MU, and planning time.

## Materials and methods

This was a retrospective study evaluating VMAT treatment plans. Patients treated between January 2026 and March 2026 were included, and data analysis was performed in May 2026. A consecutive sampling technique was used: all patients diagnosed with cervical carcinoma and treated in our Department of Radiation Oncology during the study period were considered. The sample size of 11 was determined by the number of eligible patients treated during the study window. Patients diagnosed with cervical carcinoma received treatment in our Department of Radiation Oncology, Shri Ram Murti Smarak Institute of Medical Sciences, Bareilly, Uttar Pradesh, India. The inclusion criteria were histologically confirmed cervical carcinoma, treatment with definitive external beam radiotherapy to 50 Gy in 25 fractions, and availability of a complete computed tomography (CT) simulation dataset with all target volumes and OARs contoured. The exclusion criterion was the absence of cervical carcinoma. Potential confounders included interpatient variation in bladder/rectum filling at the time of CT simulation and anatomical variation in planning target volume (PTV) and shape, both of which were not controlled for and are addressed further in the limitations. A total of 11 patients diagnosed with cervical carcinoma were included, and one-arc to four-arc VMAT plans were generated for each patient. All patients received a prescribed dose of 50 Gy in 25 fractions.

CT simulation and immobilization

All patients underwent CT simulation in the supine position using appropriate immobilization devices to ensure reproducibility during treatment delivery. Patients were instructed to maintain a comfortably filled bladder and empty rectum during simulation and treatment. CT simulation images were acquired with a 3-mm slice thickness and transferred to the Eclipse treatment planning system (TPS; version 17.0.1, Varian Medical Systems, Inc., Palo Alto, CA, USA) for contouring and treatment planning [8,9]. In recent studies of cervical cancer VMAT planning, researchers have emphasized the importance of proper immobilization and bladder preparation protocols for improving setup reproducibility and minimizing organ motion during pelvic radiotherapy [8]. Similar CT simulation protocols using supine positioning, thermoplastic immobilization, and bladder filling instructions have also been reported in recent pelvic VMAT studies [9].

Target volume and OAR delineation

The target volumes and OARs were delineated on axial CT simulation images using the Eclipse TPS (version 17.0.1). Clinical target volume and PTV were contoured according to institutional protocols and radiation oncology guidelines. Appropriate margins were applied to generate the PTV to compensate for setup uncertainties and internal organ motion.

OARs, including the bladder, rectum, bowel, pelvic bone marrow, and bilateral femoral heads, were contoured for dosimetric evaluation. Particular attention was given to minimizing radiation exposure to these critical structures while maintaining adequate target coverage. Recent studies in cervical cancer VMAT planning have demonstrated the importance of accurate target delineation and OAR contouring for improving CI and HI and reducing treatment-related toxicity [10,11]. Similar contouring approaches for pelvic targets and OARs have been reported in recent VMAT optimization studies for cervical carcinoma.

VMAT planning

For each of the 11 patients, four separate VMAT plans consisting of one, two, three, and four arcs were generated using the Eclipse TPS. All plans were created in the same CT image using identical optimization objectives, dose constraints, and prescription dose to allow for accurate dosimetric comparison among different arc arrangements. The one- to four-arc configurations are illustrated in Figures 1-4, respectively.

**Figure 1:**
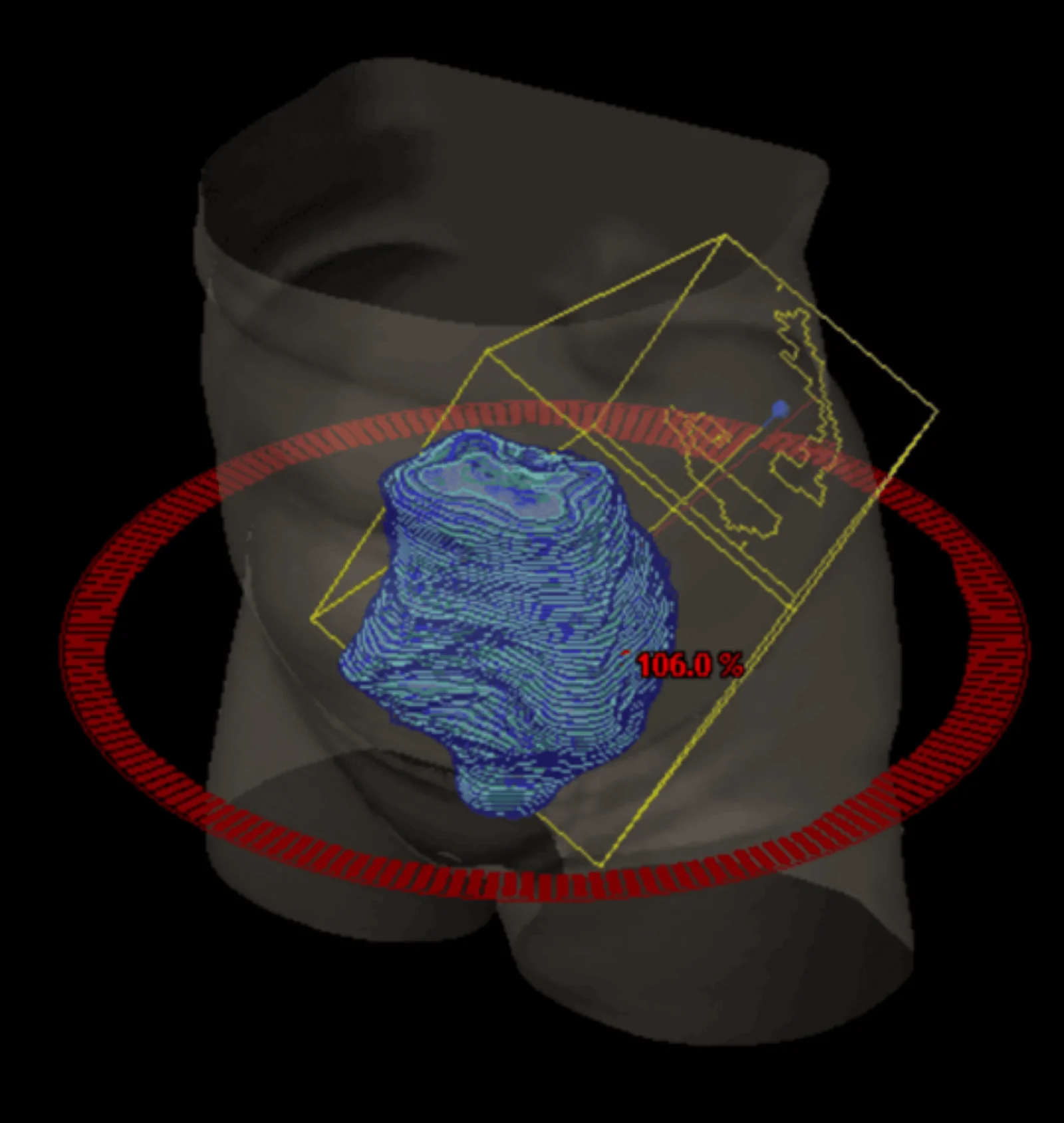
VMAT arc arrangement showing one arc as generated in the treatment planning system VMAT: volumetric modulated arc therapy

**Figure 2:**
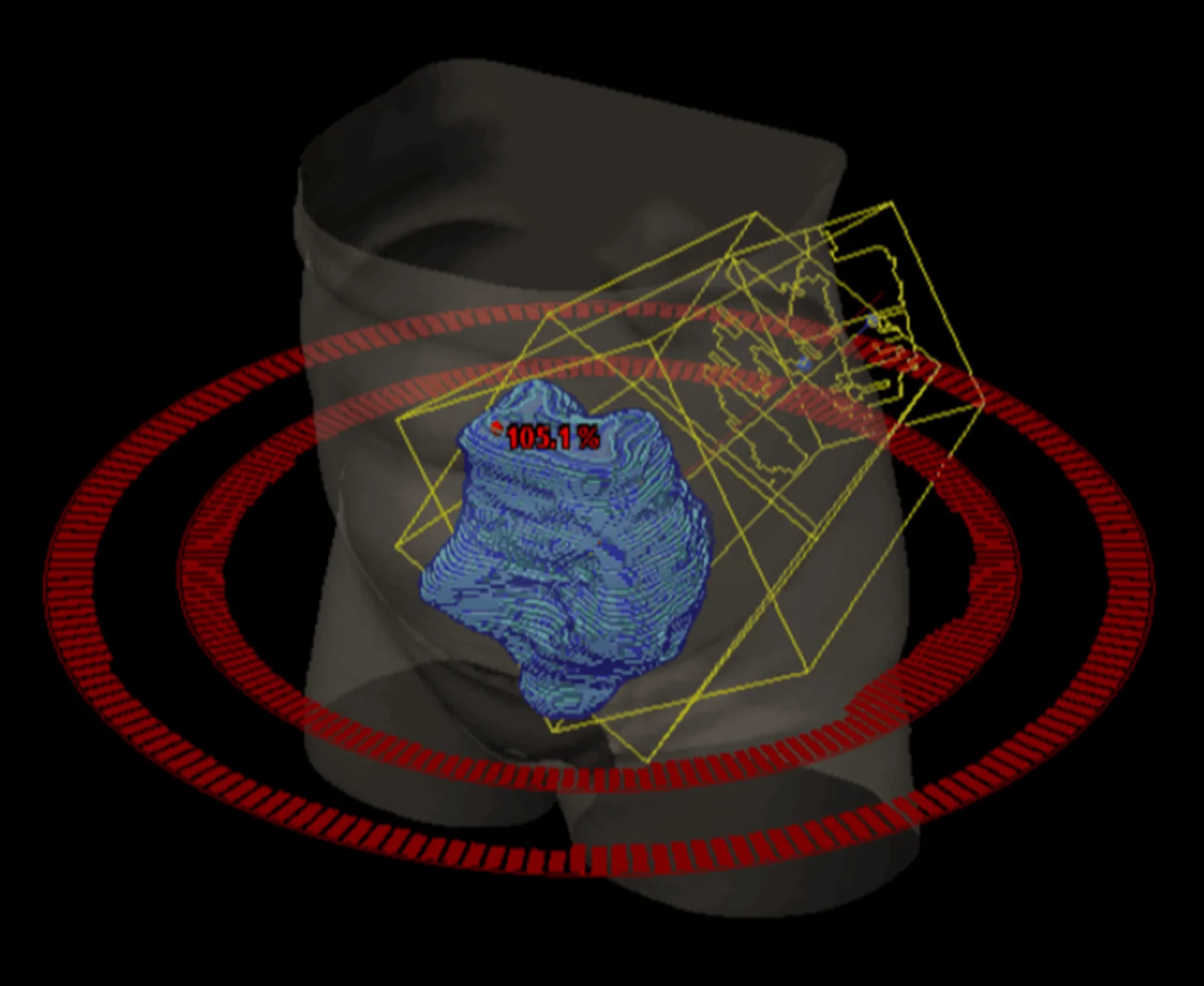
VMAT arc arrangements showing two arcs as generated in the treatment planning system VMAT: volumetric modulated arc therapy

**Figure 3:**
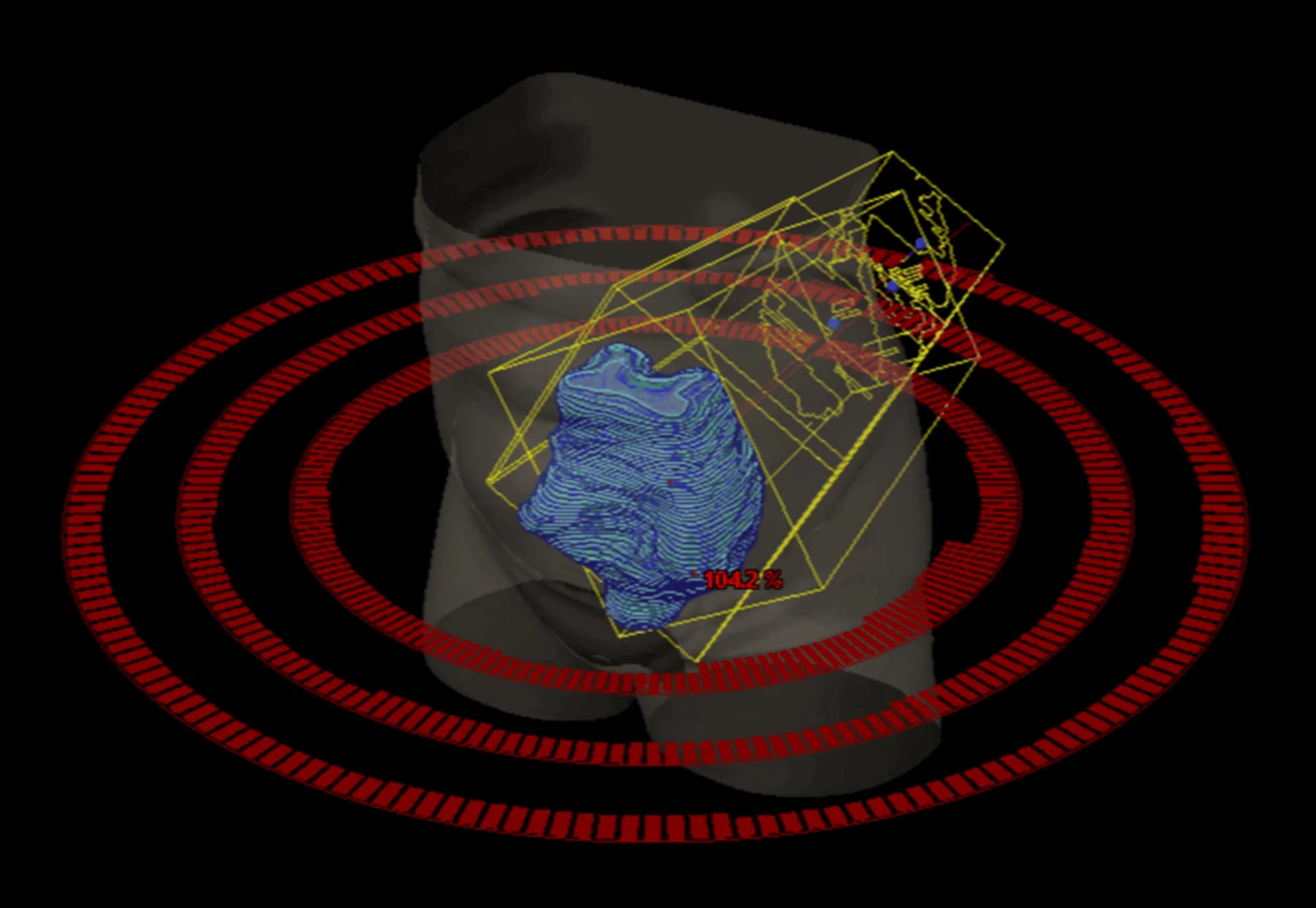
VMAT arc arrangements showing three arcs as generated in the treatment planning system VMAT: volumetric modulated arc therapy

**Figure 4:**
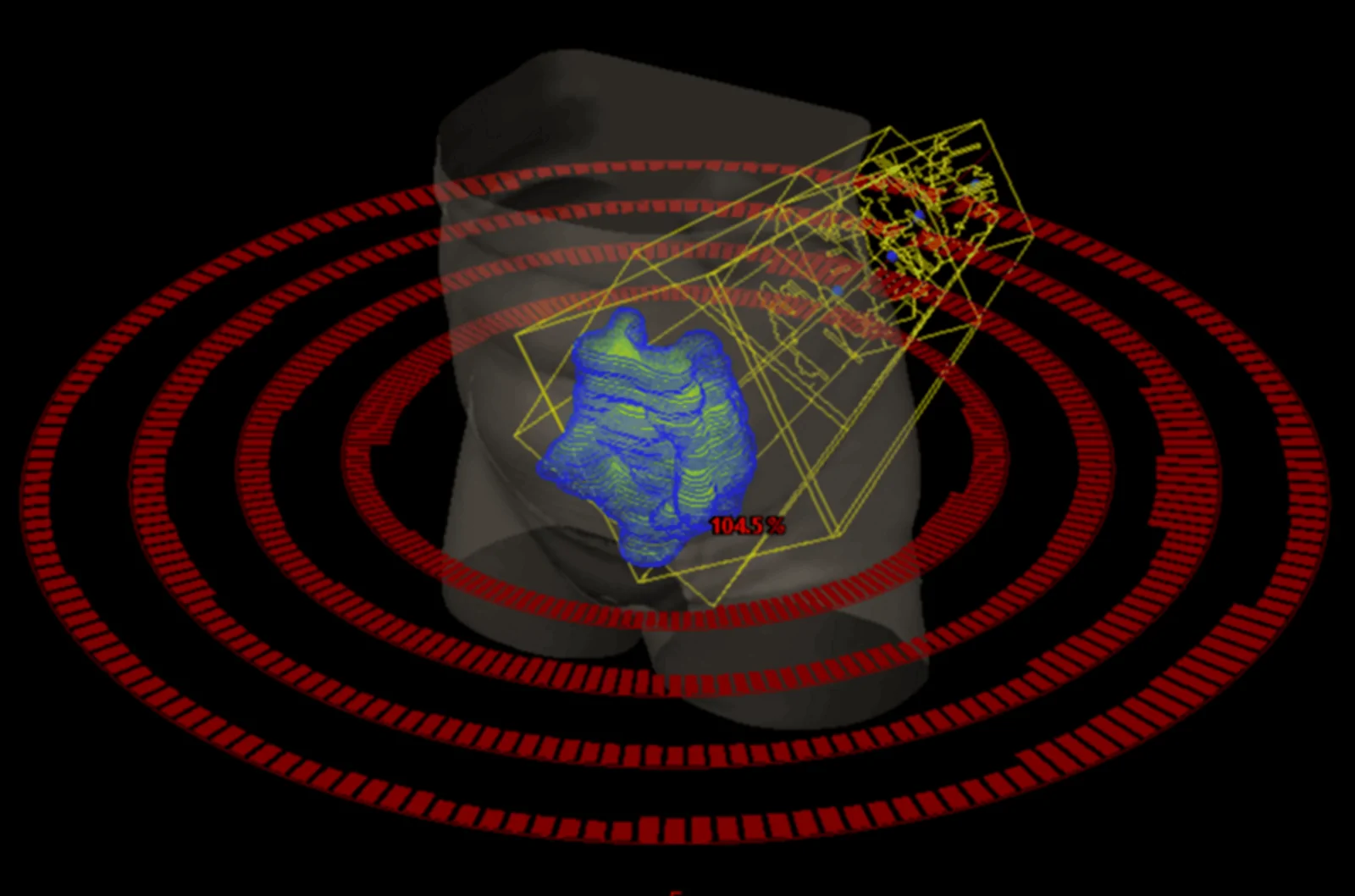
VMAT arc arrangements showing four arcs as generated in the treatment planning system VMAT: volumetric modulated arc therapy

Arc geometry

For single-arc treatment planning, the gantry rotated in the clockwise direction (181° to 179°), and the collimator angle was 30°. For Arc 2, the gantry rotated in the anticlockwise direction (179° to 181°), and the collimator angle was 330°. For Arc 3, the gantry rotated in the clockwise direction (181° to 179°), and the collimator angle was 30°. For Arc 4, the gantry rotated in the anticlockwise direction (179° to 181°), and the collimator angle was 330°.

Treatment planning was performed for a prescribed dose of 50 Gy in 25 fractions using a LINAC with 6-MV photon beam energy according to the institutional clinical protocol. During optimization, adequate target coverage and minimization of dose to surrounding OARs were prioritized. Dose optimization was performed using a photon optimizer (PO version 17.0.1; Varian Medical Systems, Inc.), while final dose calculation was carried out using the Anisotropic Analytical Algorithm (AAA version 17.0.1; Varian Medical Systems, Inc.). Beam geometry optimization was performed using the Plan Geometry Optimizer (PGO version 13.6.23; Varian Medical Systems, Inc.).

The AAA was selected because of its improved heterogeneity-corrected photon dose calculation accuracy, while the PO enabled efficient inverse planning dose modulation for VMAT delivery, which involves continuous gantry rotation around the patient with simultaneous variation of dose rate, gantry speed, and MLC motion to achieve a highly conformal dose distribution. Different arc configurations were generated to evaluate the effect of arc number on target conformity, homogeneity, MU efficiency, OAR sparing, and treatment delivery efficiency. Different arc configurations were created to evaluate the effect of arc number on target coverage, dose homogeneity, conformity and OAR sparing, MU efficiency, and treatment delivery parameters [12].

Plan evaluation

All VMAT plans were evaluated based on target coverage, dose homogeneity, conformity, OAR sparing, MU efficiency, and treatment delivery parameters. Dosimetric evaluation was performed using dose-volume histogram analysis from the Eclipse TPS. Target volume was evaluated using CI and HI. CI was used to evaluate how well the prescribed dose conforms to the target volume, while dose homogeneity was used to evaluate the dose uniformity within the target volume [6]. The CI quantifies how well the prescription dose distribution conforms to the target volume in terms of shape and size. The CI was calculated using the formula $CI_{\mathrm{Paddick}} = \frac{(TV_{\mathrm{PIV}})^2}{TV \times PIV}$. In this formula, TV_PIV_ stands for the target volume (TV) covered by the prescription isodose volume (PIV); TV denotes total target volume, and PIV denotes total prescription isodose volume. The HI is used to evaluate the homogeneity of dose distribution in the PTV. HI was calculated as $HI = \frac{D_{2\%} - D_{98\%}}{D_{50\%}}$, are the doses received by 2%, 98%, and 50% of the PTV volume, respectively. HI values close to 0 indicate greater homogeneity.

OAR dosimetric parameters (D50% and D100% for the bladder and rectum, D195cc and D100% for the bowel, mean dose for pelvic bone marrow, and Dmax for bilateral femoral heads) were recorded and compared among the one-, two-, three-, and four-arc VMAT plans [11,12]. All dosimetric parameters obtained from different arcs were statistically analyzed to determine the optimal arc arrangements for cervical carcinoma VMAT planning. Dose distribution images showing homogeneity for one- to four-arc configurations are shown in Figures 5-8, respectively.

**Figure 5:**
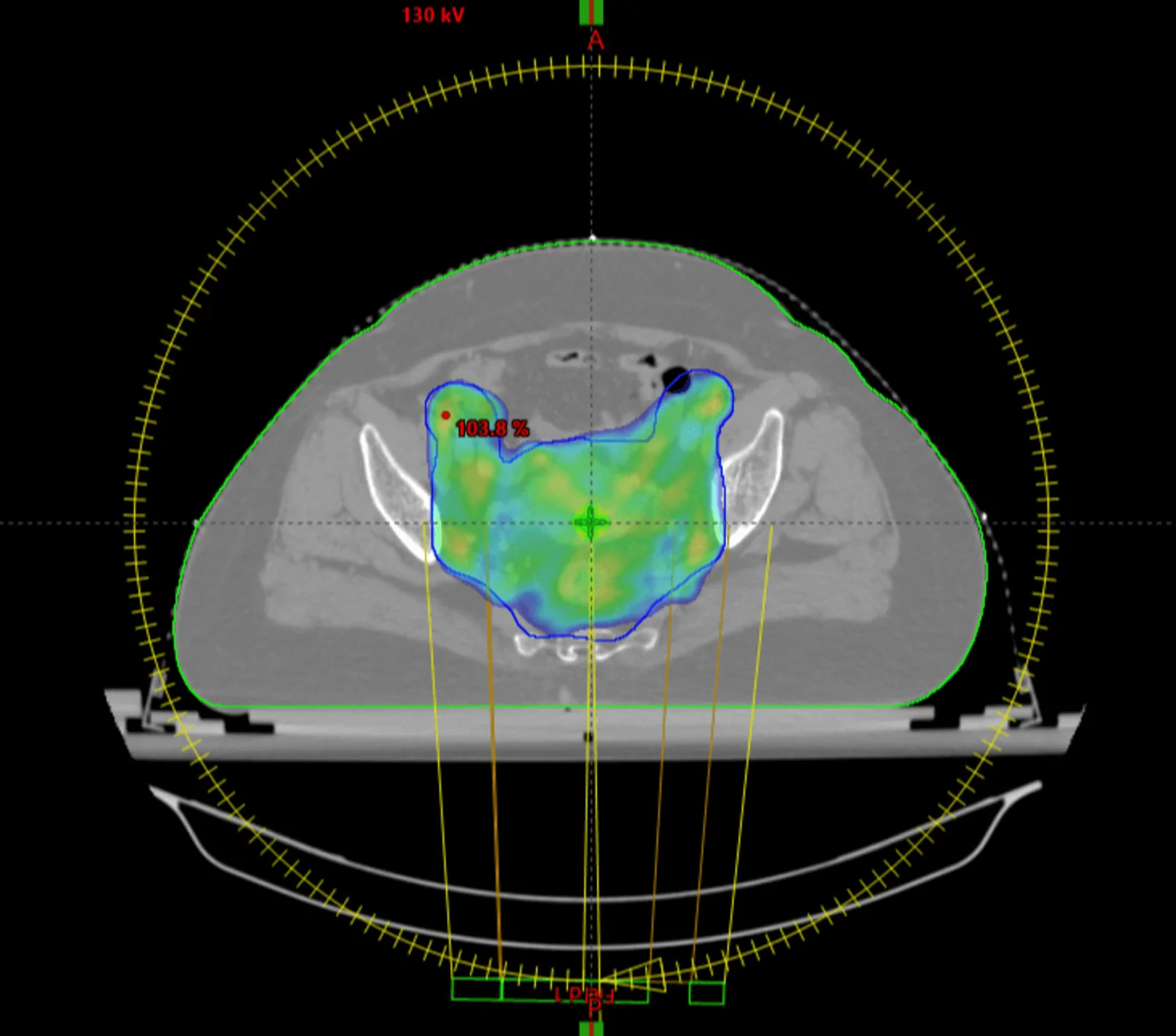
Homogeneity for Arc 1

**Figure 6:**
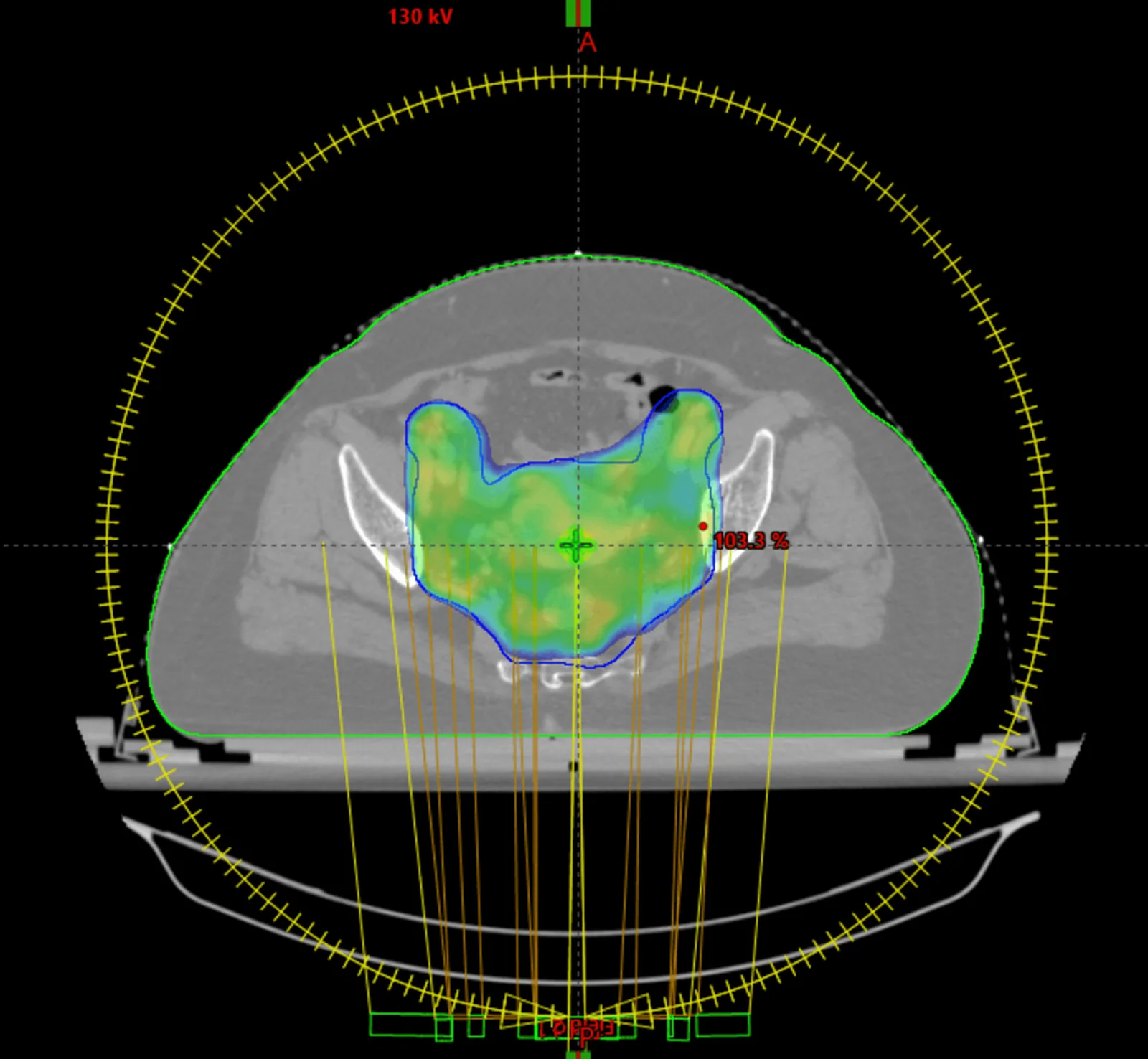
Homogeneity for Arc 2

**Figure 7:**
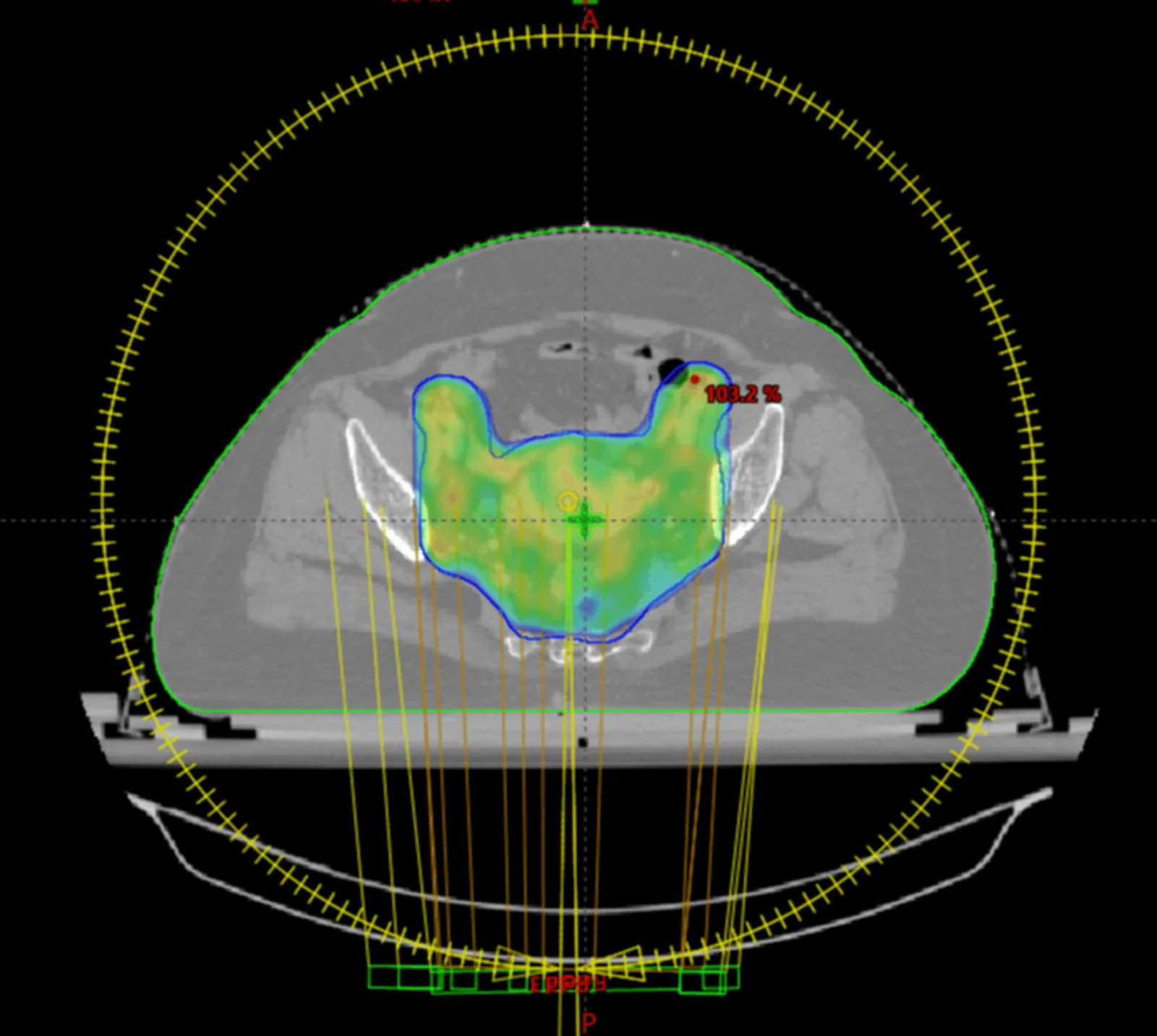
Homogeneity for Arc 3

**Figure 8:**
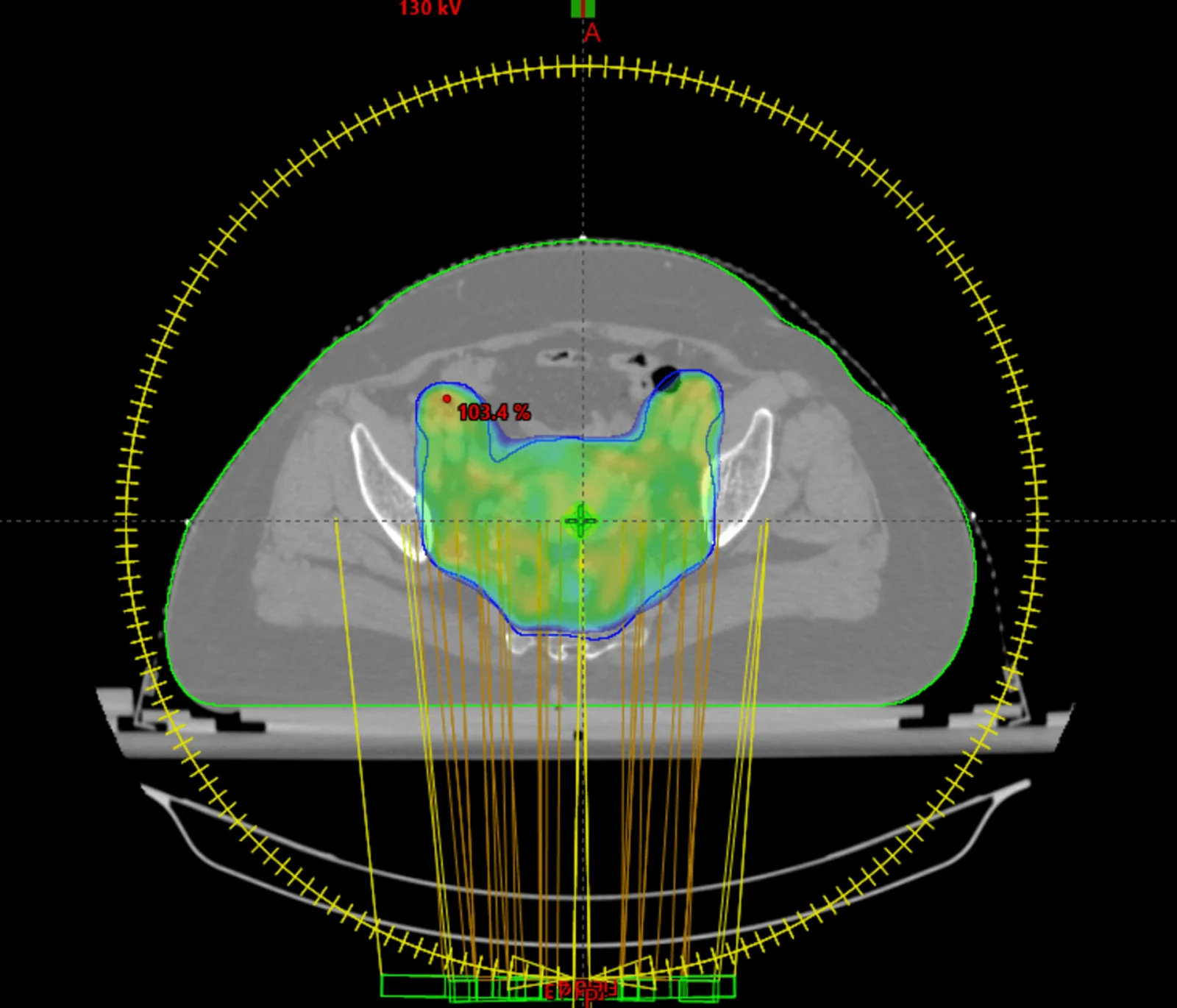
Homogeneity for Arc 4

Statistical analysis

Statistical analysis was performed using Microsoft Excel (Microsoft® Corp., Redmond, WA, USA). The number of patients was (n = 11). The four arc configurations (one, two, three, and four arcs) constituted repeated measurements on the same 11 patients. The Friedman test was used to test for an overall effect of arc number on each dosimetric and efficiency parameter, as the appropriate nonparametric omnibus test for this repeated-measures design. For parameters showing a statistically significant Friedman result (p < 0.05), post-hoc pairwise comparisons between arc configurations were performed using the Wilcoxon signed-rank test, with p-values adjusted for the six pairwise comparisons using the nonparametric analysis framework. A Holm-adjusted p-value < 0.05 was considered statistically significant. The Friedman test statistic (X^2^), with three degrees of freedom, is reported alongside each omnibus p-value in Table 1.

## Results

The conformity of the target remained stable regardless of arc number, with a median CI of 0.83 (IQR, 0.82-0.86) in all four arc configurations and no significant overall Friedman effect (X^2^ = 6.0, p = 0.112). The optimization objectives and dose constraints were identical across all plans.

HI changed significantly with arc number: median HI was 0.09 (one arc), 0.07 (two arcs), 0.06 (three arcs), and 0.06 (four arcs), indicating a significant overall effect (Friedman X^2^ = 18.692, p < 0.001). Holm-adjusted post-hoc comparisons confirmed significant differences for one-arc versus four-arc (p = 0.034) and two-arc versus four-arc (p = 0.037); no other pairwise HI comparison reached significance. Monitor units also changed significantly with arc number (median MU, 450, 449, 490, and 505 for one to four arcs; X^2^ = 22.056, p < 0.001), with significant post-hoc differences for one versus three (p = 0.037), one versus four (p = 0.023), and two versus four (p = 0.023). Bone marrow Dmean decreased significantly with arc number (median, 32.90, 32.60, 32.40, and 32.30 Gy for one to four arcs; X^2^ = 16.879, p < 0.001), with significant post-hoc reductions for one versus four (p = 0.036) and two versus four (p = 0.046). Bladder D100%, bowel D195cc, bilateral femoral head Dmax, and planning time each showed a statistically significant overall Friedman effect (p < 0.05; Table 1), but after Holm correction, only the bladder D100% three-arc versus four-arc comparison remained significant (p = 0.023). No other pairwise comparison for these parameters survived correction, so they should be interpreted as an overall arc-number effect without a specific identifiable pairwise driver. Bladder D50%, rectum D50%, rectum D100%, bowel D100%, and CI showed no significant overall Friedman effect. The dosimetric parameters are summarized in Table 1. 

**Table 1:** Dosimetric comparison of different parameters Values are median (IQR). Overall comparison was performed using the Friedman test; df = 3. A p < 0.05 indicates statistical significance.

Parameter	Arc 1, Median (IQR)	Arc 2, Median (IQR)	Arc 3, Median (IQR)	Arc 4, Median (IQR)	Friedman χ²	df	p-value
Homogeneity Index	0.09 (0.08-0.09)	0.07 (0.07-0.08)	0.06 (0.06-0.07)	0.06 (0.06-0.07)	18.692	3	<0.001
MU	450.00 (378.50-461.00)	449.00 (427.00-487.50)	490.00 (417.50-532.00)	505.00 (442.00-559.50)	22.056	3	<0.001
Bladder D50%	48.10 (48.00-48.85)	48.10 (46.90-48.95)	48.00 (46.55-48.90)	48.10 (46.25-48.95)	5.717	3	0.126
Bladder D100%	29.00 (28.19-32.60)	29.90 (27.05-34.40)	30.10 (28.45-35.30)	28.40 (25.90-32.10)	11.278	3	0.01
Rectum D50%	45.90 (44.00-47.95)	44.60 (44.10-46.65)	44.30 (43.20-46.65)	43.80 (42.15-45.30)	7.33	3	0.062
Rectum D100%	23.40 (11.30-24.65)	20.20 (12.35-23.60)	21.10 (13.55-26.65)	22.20 (10.55-23.80)	1.917	3	0.59
Bowel D195 cc	30.00 (27.30-33.85)	29.40 (26.90-33.60)	29.50 (27.25-32.85)	28.80 (25.30-32.45)	8.771	3	0.032
Bowel D100%	1.75 (1.41-2.40)	1.80 (1.35-2.90)	1.90 (1.30-3.65)	2.10 (1.54-3.10)	3.686	3	0.297
Bone Marrow Dmean	32.90 (32.35-35.40)	32.60 (32.15-33.35)	32.40 (31.55-33.00)	32.30 (31.40-32.90)	16.879	3	<0.001
LT Femur Dmax	44.80 (43.39-45.70)	44.10 (43.55-45.00)	44.50 (43.40-45.25)	44.00 (42.90-45.05)	8.126	3	0.043
RT Femur Dmax	44.60 (43.95-45.28)	44.10 (43.45-44.35)	43.80 (43.25-44.15)	43.70 (42.95-44.40)	8.692	3	0.034
Conformity Index	0.83 (0.82-0.86)	0.83 (0.82-0.86)	0.83 (0.82-0.86)	0.83 (0.82-0.86)	6	3	0.112
Planning time, sec	920 (800-972)	873 (789-939.5)	909 (856.5-952)	965 (860-1018)	8.127	3	0.043

HI stood out as the most responsive parameter to arc number, with the clearest and most robust omnibus and post-hoc significance of any parameter tested. CI showed no significant change across arc configurations (Table 1), consistent with conformity being governed primarily by the shared planning objectives applied identically across all plans. Post-hoc pairwise comparisons (Wilcoxon signed-rank test, Holm-adjusted) are presented in Table 2, restricted to the eight parameters for which the overall Friedman test was significant.

**Table 2:** Post-hoc pairwise comparisons We used only the outcomes where the overall Friedman test was significant. p-values were adjusted using the Holm method following paired Wilcoxon signed-rank tests. Bold values indicate Holm-adjusted p < 0.05.

Parameter	Arc 1 vs 2	Arc 1 vs 3	Arc 1 vs 4	Arc 2 vs 3	Arc 2 vs 4	Arc 3 vs 4
Homogeneity Index	0.073	0.769	0.034	0.769	0.037	0.544
Monitor Unit (MU)	0.166	0.037	0.023	0.166	0.023	0.088
Bladder D100%	1.000	0.672	1.000	1.000	0.280	0.023
Bowel D195 cc	0.280	0.087	0.280	1.000	1.000	1.000
Bone Marrow Dmean	0.442	0.199	0.036	0.120	0.046	0.442
LT Femur Dmax	0.413	0.691	0.336	0.691	0.691	0.691
RT Femur Dmax	0.673	0.673	0.087	0.094	0.951	0.951
Planning Time	0.824	0.500	0.569	0.197	0.569	0.569

## Discussion

This study clearly demonstrates that arc number in VMAT planning for cervical carcinoma matters for dose homogeneity, monitor units, and bone marrow dose, with four-arc plans showing the strongest statistical support for these three parameters, although conformity remains stable because the dose constraints are the same for all arc plans created. The four-arc configuration consistently provides the most favorable dosimetric profile. This study of 11 patients provides a well-researched hypothesis that can guide both institutional practice and future prospective research. VMAT planning and dosimetric analyses for cervical cancer have been documented in recent studies assessing treatment planning optimization and dosimetric results [11,12].

The conformity of 0.83 (IQR, 0.82-0.86), identical across all four arc configurations with no significant overall Friedman effect (p = 0.112), is consistent with the findings of Yadav et al. [4], who compared single-arc RapidArc with fixed-field IMRT in cervical cancer patients. They found uncompromised target coverage with arc-based delivery, as well as significantly reduced integral dose to healthy tissue, and achieved better sparing of the bladder and rectum compared with IMRT. Agrawal et al. [5], comparing 3D conformal radiation therapy with VMAT in locally advanced cervical cancer, further confirmed that VMAT achieves superior target conformity over conventional techniques. Regarding the stability of CI for the predominantly convex volumes encountered in cervical cancer, a single full arc provides sufficient conformity of the prescription isodose tightly around the PTV. Clinicians can confidently adopt a three- or four-arc configuration without concern for target coverage. In our study, extending the findings of Yadav et al. [4], who compared arc therapy with IMRT as two different techniques, our data go a step further by demonstrating that even within VMAT itself, increasing the arc number does not affect conformity, an observation not previously reported in cervical carcinoma planning.

The improvement in dose homogeneity with additional arcs is the most clearly supported finding in our study and the supporting literature. Median HI decreased from 0.09 (one arc) to 0.06 (four arcs), representing a statistically significant overall effect (Friedman p < 0.001), with Holm-adjusted post-hoc comparisons confirming significant differences for one-arc versus four-arc (p = 0.034) and two-arc versus four-arc (p = 0.037); no other pairwise HI comparison reached significance. Harijith et al. [12], comparing one-, two-, three-, and four-arc VMAT plans in 20 head and neck cancer patients, reported a statistically significant progressive improvement in HI with increasing arc number. Our findings, now confirmed using the appropriate repeated-measures (Friedman) test for this study design, align closely with theirs. Otto and Clark [13] demonstrated that rotational intensity-modulated delivery techniques improve dose modulation and treatment efficiency by increasing angular sampling around the target volume, a pattern that fits our finding for pelvic cancer. Guy et al. [6], whose cervical cancer planning study compared RapidArc with IMRT, demonstrated that arc-based delivery achieved significantly better HI than fixed-field techniques, and our data extend this logic within arc therapy itself. This demonstrates that four-arc plans outperform single-arc plans in the same way that VMAT as a whole outperforms IMRT.

It is important to note that an HI reduction from 0.09 to 0.06 (median) may be clinically relevant. The PTV for cervical carcinoma is geometrically heterogeneous and encompasses a large primary tumor volume alongside regional lymph node stations at varying distances from the isocenter. A single arc may struggle to deliver uniformly to this spread-out target, creating cold spots in distant nodal regions and hot spots near the isocenter. Over 25 fractions, an HI of 0.09 indicates greater dose heterogeneity within the PTV than an HI of 0.06, while adjacent normal tissue may receive additional dose. Reducing the HI to 0.06 with a four-arc plan meaningfully improves dose uniformity, with potential implications for both nodal control and late tissue tolerance. This mechanistic reasoning is supported by clinical outcomes data. In a prospective study of 72 patients with locally advanced cervical cancer treated with VMAT followed by 3D image-guided brachytherapy, Van Anh et al. [14] reported high control rates with manageable toxicity outcomes that the authors attributed in part to the dose homogeneity achieved by arc-based planning.

For our OARs, doses showed consistent, clinically plausible reductions across all matrices as arc number increased, even though none reached statistical significance. The rectum D50% reduction (median, 45.90 Gy for one arc to 43.80 Gy for four arcs) approached but did not reach overall statistical significance (Friedman p = 0.062). Late rectal toxicity is one of the most significant late effects of pelvic radiotherapy, and the dose-response relationship for the rectum is among the best characterized in the field. Ong et al. [15], fitting Lyman-Kutcher-Burman normal tissue complication probability models prospectively, scored late toxicity data from 150 pelvic cancer patients treated with VMAT and demonstrated that intermediate rectal dose-volume parameters are the strongest predictors of late grade 2+ GI toxicity, parameters that our four-arc plans consistently minimize. Similarly, Zimmermann et al. [16], comparing VMAT with conventional planning for rectal cancer chemotherapy, found that VMAT planning significantly reduced late sphincter toxicity compared with conventional techniques and emphasized that rectal dose-volume constraints were the key modifiable factor. Our data suggest that, within VMAT planning, cervical arc number is an additional factor for reducing rectal intermediate-dose exposure and one that adds no planning complexity beyond choosing an additional arc at the time of plan setup.

The bone marrow Dmean decreased significantly with arc number (median, 32.90 Gy for one arc to 32.30 Gy for four arcs; Friedman p < 0.001), with Holm-adjusted post-hoc comparisons confirming significant reductions for one-arc versus four-arc (p = 0.036) and two-arc versus four-arc (p = 0.046). This study did not involve or track chemotherapy administration for any patient; cisplatin is referenced below only because it featured in the concurrent chemoradiotherapy regimens described in the cited literature, as general background on why pelvic bone marrow dose is a clinically relevant endpoint, not because cisplatin was used in this study. As this study did not collect chemotherapy dose/schedule, cycle count, baseline hematological parameters, or treatment interruption data for its own patients, whether this reduction would translate into reduced hematologic toxicity or improved chemotherapy compliance cannot be determined from this dosimetric study. Wang et al. [17] demonstrated in a prospective randomized controlled trial that single-photon emission CT-defined active bone marrow-sparing VMAT significantly reduced grade 3+ hematological toxicity and improved cisplatin delivery compared with standard VMAT, confirming the direct clinical translation of bone marrow dose reduction. Konnerth et al. [18] found in a systematic review of published dose constraints that pelvic bone marrow V20% < 86.6% and V40 < 40% were the most reproducible predictors of hematological toxicity reduction across studies. By consistently achieving lower bone marrow Dmean, our four-arc plans are better positioned to satisfy these constraints than a one-arc configuration, producing a practical advantage that could translate into fewer cisplatin dose reductions and better overall treatment. Kuipers et al. [19], using Pareto front analysis in 20 cervical cancer patients, further demonstrated that meaningful bone marrow dose reductions are achievable in VMAT planning without proportionately increasing rectal or bladder doses, a reassuring finding that aligns with our study, in which OAR reductions with one-arc plans are seen simultaneously across multiple organs rather than at the expense of one another. Huang et al. [20], in a prospective randomized trial of pelvic bone marrow-sparing radiotherapy in cervical cancer, confirmed that reducing bone marrow doses during chemotherapy significantly reduced hematologic toxicity, providing indirect clinical support for the direction of our now-confirmed finding.

In the present study, bowel V195cc also reached overall significance (X^2^ = 8.771, p = 0.032), with a progressive numerical reduction from a median of 30.00 Gy at one arc to 28.80 Gy at four arcs. However, none of the individual pairwise comparisons survived Holm correction, indicating that the overall trend across arc numbers is real, but that no single arc-to-arc step can be isolated as the source of the benefit in this cohort. This is directionally consistent with the field optimization result of Zeng et al. [7], who found that split-field four-arc VMAT configurations significantly reduced bowel exposure in cervical cancer patients with inguinal lymphatic drainage extension. The modified fixed-field VMAT approach of Jindakan et al. [21], which significantly reduced OAR doses, including bowel dose, by optimizing jaw position in cervical cancer planning, further supports the principle that any modification that gives the optimizer more geometric freedom, whether through arc number, field geometry, or jaw configuration, tends to produce bowel dose benefits in pelvic VMAT targets, even when individual pairwise arc comparisons do not reach significance.

The MUs rose significantly with arc number overall (X^2^ = 22.056, p < 0.001), with a median increase from 450 MU at one arc to 505 MU at four arcs. Post-hoc testing showed that this was driven by comparisons involving the extremes of arc number: one-arc versus three-arc (p = 0.037), one-arc versus four-arc (p = 0.023), and two-arc versus four-arc (p = 0.023) were all significant, while one-arc versus two-arc, two-arc versus three-arc, and three-arc versus four-arc were not. This indicates that MU accumulates gradually across arcs but only becomes a statistically distinguishable burden once the plan reaches three or four arcs relative to one or two. The MU data from our study sit comfortably within published ranges for VMAT in cervical cancer. Huang et al. [22], comparing two VMAT optimization algorithms in 34 cervical cancer patients, reported MU values in the range of 400-650 MU for dual-arc plans, broadly consistent with our four-arc median of 505 MU. Their study also demonstrated that MU output is strongly influenced by optimization algorithms and jaw configuration, not just arc number, which provides context for why the MU differences in our study, although statistically significant by Friedman and Holm-adjusted post-hoc testing, were modest in absolute magnitude. The 12.2% MU increase from one-arc to four-arc planning in our cohort is clinically acceptable. For comparison, Jindakan et al. [21] reported a statistically significant MU increase with modified VMAT but showed that beam-on time was simultaneously reduced, illustrating that MU alone is not a sufficient measure of delivery efficiency and that arc-time relationships depend on gantry speed modulation as much as on MU output.

Comparing our VMAT study directly with Thakur et al. [23], who compared VMAT with IMRT in 30 cervical cancer patients undergoing chemotherapy, we found that VMAT achieved better bone marrow sparing and comparable target coverage, a finding that aligns with our four-arc data showing the lowest bone marrow Dmean in the study. Importantly, the CI values in their VMAT study (approximately 0.83-0.85) and OAR dose ranges are closely comparable to those of our four-arc plan, supporting the validity of our findings relative to a published study. Yang et al. [24], reporting a single-institution experience of 102 VMAT-treated patients with locally advanced cervical cancer, achieved a five-year local control rate of 89.3%, with only 3% grade ≥ 3 late rectal toxicity, outcomes that the authors attributed to careful adherence to rectal dose-volume constraints. Our four-arc plans, with rectum D50% of 43.80 (42.15-45.30) Gy, are better positioned to meet these constraints than our one-arc plans, with 45.90 (44.00-47.90) Gy. The prospective Vietnamese cohort study by Van Anh et al. [14] similarly reported manageable toxicity using dual-arc VMAT, which resulted in acceptable clinical outcomes. The active bone marrow-sparing trial of Wang et al. [17] is the most direct clinical parallel to our dosimetric finding. They showed that reducing bone marrow Dmean by modifying VMAT arc geometry reduced grade 3+ hematologic toxicity from 35% to 16% (p < 0.05) and improved cisplatin completion rates, providing direct clinical validation for the dosimetric direction toward which our four-arc data point.

Planning time varied with arc number, ranging from a median of 14:33 minutes for two-arc plans to 16:05 minutes for four-arc plans, and this overall difference reached statistical significance on Friedman testing (p = 0.043). However, none of the individual pairwise comparisons between arc configurations remained significant after Holm correction, indicating that the time cost of additional arcs is measurable at the group level but cannot be attributed with confidence to any single arc-number comparison. The absolute difference between the fastest and slowest configurations was under two minutes, which is modest in the context of the radiotherapy planning workflow and should not be a deterrent to adopting four-arc plans in routine practice. Jindakan et al. [21], who demonstrated increased planning complexity with modified VMAT geometry in cervical cancer, similarly concluded that marginal increases in planning burden were justified by the dosimetric gain achieved. More clinically relevant time considerations in VMAT optimization include algorithm choice, MU output, and delivery efficiency, which are influenced more by algorithm choice and jaw configuration than by arc number alone. This finding contextualizes the MU increase noted above and suggests that delivery time would not scale proportionately with planning time even as arc number rises.

The bilateral femoral head doses showed a consistent decrease with increasing arc number, with median left femoral head Dmax decreasing from 44.80 Gy (one arc) to 44.00 Gy (four arcs) and median right femoral head Dmax decreasing from 44.60 Gy to 43.70 Gy across one-arc to four-arc plans; both reached overall statistical significance on Friedman testing (left femur, p = 0.043; right femur, p = 0.034), although no individual pairwise comparison remained significant after Holm correction. Importantly, no arc configuration produced femoral head doses approaching the standard tolerance threshold, and femoral head dose did not increase with additional arcs in any case in the dataset. This confirms that the additional angular sampling provided by three-arc and four-arc configurations redistributes dose more widely across pelvic tissue without concentrating dose in adjacent bony structures. Azalmad et al. [25] similarly demonstrated improved OAR sparing with increasing arc number in cervical cancer VMAT.

The findings of our study lead to a clear institutional recommendation. For VMAT planning in cervical carcinoma, the four-arc configuration offers the most favorable overall dosimetric profile, with the best homogeneity, lowest OAR doses across all measured structures, and femoral head doses that remain within safe limits throughout. This conclusion is supported by Azalmad et al. [25]. The planning time cost is small and clinically acceptable. Where departmental workflow constraints favor a compromise, three-arc plans represent a reasonable alternative, offering dosimetric parameters intermediate between single-arc and four-arc configurations without a proportionate increase in planning burden. Single-arc planning, while adequate for target conformity, consistently produced the least favorable homogeneity and OAR sparing across this cohort and should be regarded as the minimum acceptable rather than the preferred configuration for pelvic VMAT in cervical cancer.

Limitations

This study has several limitations. Eleven patients provide adequate proof of concept, but insufficient statistical power for OAR comparisons, and the originally reported six uncorrected pairwise t-tests per parameter risked false positives. This has been resolved by re-analysis using the Friedman test with Holm-adjusted Wilcoxon signed-rank post-hoc comparisons, as reported in Tables 1-2. All plans were generated by a single planner on one TPS platform, and results may differ across centers, planners, and TPS. Only planning time was measured, not beam-on/delivery time; we did not report body V5, V10, V20, integral dose, or other low-dose-bath metrics, so the possibility that additional arcs increase low-dose spread to normal tissue even while reducing high-dose OAR volumes cannot be excluded. Explicit target coverage metrics (PTV V95%, D95%, D98%, D2%) were not reported beyond CI/HI. No clinical toxicity or tumor-control data were collected, so this study cannot establish that four-arc VMAT is clinically optimal, only that it shows favorable dosimetric trends in this small cohort.

The study is purely dosimetric; whether the improvements observed translate into better clinical outcomes requires prospective investigation with larger patient numbers. Identical optimization objectives were applied across all four arc configurations, which may slightly disadvantage or advantage specific arc arrangements compared with configuration-specific objective tuning. 

## Conclusions

This study demonstrates that increasing arc number in VMAT planning for cervical carcinoma from one to four arcs significantly improves dose homogeneity and reduces bone marrow Dmean while maintaining stable target conformity, based on Friedman testing with Holm-adjusted post-hoc comparisons. This benefit comes at the cost of a significant increase in monitor units. Bladder D100%, bowel D195cc, femoral head dose, and planning time each showed a significant overall arc-number effect, although, apart from the bladder D100% three-versus-four-arc comparison, no specific pairwise comparison survived correction. Four-arc plans offer the most statistically supported dosimetric profile; three-arc planning offers a justifiable alternative in settings where planning resources are constrained.
